# Body Mass Index: Accounting for Full Time Sedentary Occupation and 24-Hr Self-Reported Time Use

**DOI:** 10.1371/journal.pone.0109051

**Published:** 2014-10-08

**Authors:** Catrine Tudor-Locke, John M. Schuna, Peter T. Katzmarzyk, Wei Liu, Karen S. Hamrick, William D. Johnson

**Affiliations:** 1 Pennington Biomedical Research Center, Baton Rouge, Louisiana, United States of America; 2 U.S. Department of Agriculture Economic Research Service, Washington, DC, United States of America; McGill University, Canada

## Abstract

**Objectives:**

We used linked existing data from the 2006–2008 American Time Use Survey (ATUS), the Current Population Survey (CPS, a federal survey that provides on-going U.S. vital statistics, including employment rates) and self-reported body mass index (BMI) to answer: How does BMI vary across full time occupations dichotomized as sedentary/non-sedentary, accounting for time spent in sleep, other sedentary behaviors, and light, moderate, and vigorous intensity activities?

**Methods:**

We classified time spent engaged at a primary job (sedentary or non-sedentary), sleep, and other non-work, non-sleep intensity-defined behaviors, specifically, sedentary behavior, light, moderate, and vigorous intensity activities. Age groups were defined by 20–29, 30–39, 40–49, and 50–64 years. BMI groups were defined by 18.5–24.9, 25.0–27.4, 27.5–29.9, 30.0–34.9, and ≥35.0 kg/m^2^. Logistic and linear regression were used to examine the association between BMI and employment in a sedentary occupation, considering time spent in sleep, other non-work time spent in sedentary behaviors, and light, moderate, and vigorous intensity activities, sex, age race/ethnicity, and household income.

**Results:**

The analysis data set comprised 4,092 non-pregnant, non-underweight individuals 20–64 years of age who also reported working more than 7 hours at their primary jobs on their designated time use reporting day. Logistic and linear regression analyses failed to reveal any associations between BMI and the sedentary/non-sedentary occupation dichotomy considering time spent in sleep, other non-work time spent in sedentary behaviors, and light, moderate, and vigorous intensity activities, sex, age, race/ethnicity, and household income.

**Conclusions:**

We found no evidence of a relationship between self-reported full time sedentary occupation classification and BMI after accounting for sex, age, race/ethnicity, and household income and 24-hours of time use including non-work related physical activity and sedentary behaviors. The various sources of error associated with self-report methods and assignment of generalized activity and occupational intensity categories could compound to obscure any real relationships.

## Introduction

Questionnaires typically capture domain-specific activity (e.g., leisure time physical activity, occupational activity) which presumes that these are the only activities worth tracking. Objective monitoring protocols typically direct respondents to take body worn sensors off at night [1] and there is evidence that they are frequently taken off early and/or put on late [2], resulting in lengthy segments of unaccounted time. Until now, an opportunity to account for activities during the entire 24-hour day has been missing from large-scale surveys.

The American Time Use Survey (ATUS) queries primary activities performed over the course of a 24-hour day (1,440 minutes) and allows for a full accounting of time spent in work, sleep, and other non-work, non-sleep behaviors. We have produced a crosswalk to assign metabolic equivalent (MET; 1 MET is the metabolic cost of quietly resting or ≅3.5 mL of oxygen uptake per kg body weight per minute) values to these categorized behaviors [3], specifically identifying each ATUS primary activity as a sedentary behavior, or a light, moderate, or vigorous intensity activity. This process included linking summary MET values to generalized occupational categories in an attempt to better characterize the intensity of time originally captured simplistically as “at work” in the ATUS. Of particular interest is the fact that sedentary occupations (i.e., those characterized by much sitting) have a major bearing on objectively monitored physical activity levels [4]. We have previously shown that workers employed in full time sedentary occupations were actually sedentary for approximately 11 hours per day, leaving little time to achieve recommended levels of physical activity for overall health [5]. Since the prevalence of sedentary occupations (and associated weight gain) has increased in recent decades [6], there is a growing interest in examining the impact of sedentary occupations on body habitus, taking into account behaviors enacted outside of working hours, especially for full time workers with limited personal time [7], [8], [9].

There are only a finite number of minutes in a day and the different obligations and choices about how to spend each minute likely have a complex and profound impact on BMI. The final piece of the “BMI-time use” puzzle was potentially provided when, from 2006 to 2008, the ATUS rolled out an “Eating & Health” (EH) module which included a query of self-reported height and weight, necessary for computing BMI. Therefore, this analysis combined the 2006–2008 ATUS with corresponding occupational codes from the Current Population Survey (CPS, a federal survey that provides on-going U.S. vital statistics, including employment rates) and self-reported BMI from the EH module to answer the following question: How does BMI vary across full time occupations dichotomized as sedentary/non-sedentary, with and without accounting for time spent in sleep, other sedentary behaviors, and light, moderate, and vigorous intensity activities?

## Methods

### Source data sets

The publically available 2006–2008 ATUS data along with the corresponding CPS and EH module data were downloaded, extracted, and linked (using data source specified identifiers). Details about the CPS are located at and those related to the ATUS and EH are at. A brief description of each source data set follows. A subsample of individuals (age 15 and older) drawn from households that completed the CPS in the preceding 2–5 months formed the ATUS response sample. Individuals selected from the CPS for the ATUS are routinely interviewed using a computer-assisted telephone-interviewing (CATI) system on a designated day about their use of time over the previous 24-hour day. Subsequently, each reported activity is assigned a 6-digit code guided by software that organizes and broadly classified activities into more specific categories using a hierarchical 2-digit tier system. As indicated above, we have exploited this tier-based system to assign MET values to each category [3] and have published the operative SAS program at http://riskfactor.cancer.gov/tools/atus-met/. ATUS respondents routinely report time worked at their “primary job,” however, the linked “primary occupation” that they are employed in must be retrieved using links to their corresponding and previously collected CPS data. To be clear, ATUS captures time spent at primary jobs and these can be linked to CPS records of primary occupations. We used the summary MET values for reported occupations [3] and also the more detailed values [10] available at http://riskfactor.cancer.gov/tools/ocs-met/. We have previously reported that application of the more detailed values reduces the prevalence of reported sedentary occupations and increases those with more vigorous intensity occupations. The summary values are considered a more conservative approach to estimating overall energy expenditure of occupational categories [10]. The corresponding EH module (details available at http://ers.usda.gov/data-products/eating-and-health-module-(atus) and http://www.bls.gov/tus/) was only deployed from 2006–2008, thus defining the survey window (and eligible sample size) for the analysis of these combined data sets.

### Sample description

**Figure 1:**
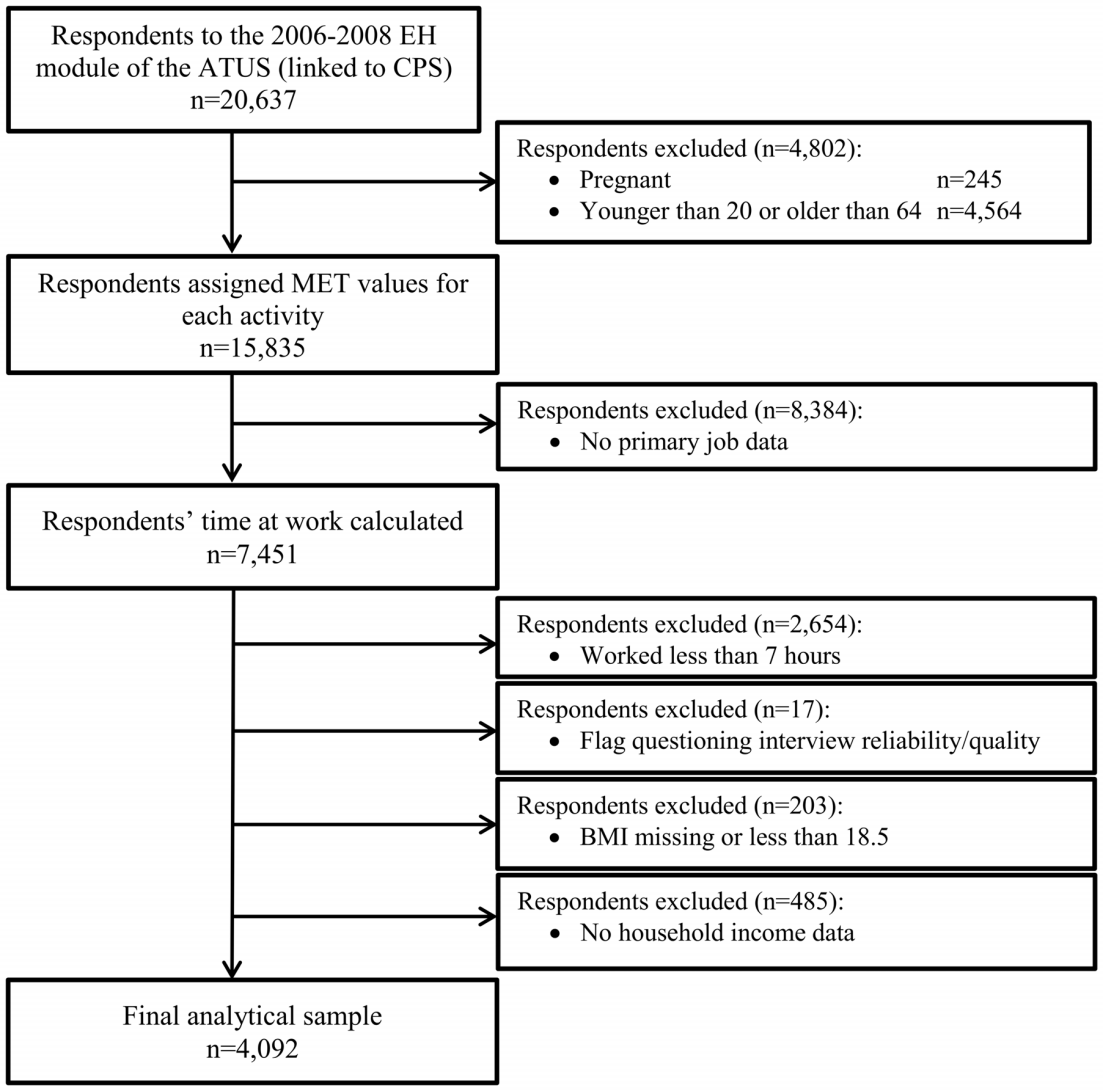
Flow chart summarizing the number of survey respondents excluded with implementation of each decision rule culminating in the final analytical data set (n = 4,092). Notes: EH = Eating and Health; ATUS = American Time Use Survey, CPS = Current Population Survey.

There were 20,637 respondents to the 2006–2008 EH module of the ATUS. The analysis sample included individuals who were 20–64 years of age (inclusively), had a CPS occupation code assigned to their primary job, reported working more than 7 hours (a proxy indicator of full time work [10]) at their primary jobs on their designated time use reporting day, and had replete height and weight fields needed to calculate BMI values. Data were excluded from respondents who reported that they were pregnant, had a BMI<18.5 kg/m^2^ or incomplete household income information, or for whom the original interviewer questioned the reliability of the interview immediately following its completion. Figure 1 is a flow chart summarizing the number of survey respondents excluded with implementation of each decision rule culminating in the final analytical data set of 4,092 individuals.

### Data treatment

Age obtained from the ATUS was categorized into groups defined by 20–29, 30–39, 40–49, and 50–64 years of age. BMI obtained from the linked EH was categorized into groups defined by 18.5–24.9, 25.0–27.4, 27.5–29.9, 30.0–34.9, and ≥35.0 kg/m^2^. Reported race/ethnicity queried in the linked CPS was recoded as White only-Hispanic, White only-non-Hispanic, Black only-Hispanic, Black only-non-Hispanic, Asian only, and Other. Household income (also from the linked CPS) was categorized as <$25,000, $25,000–49,000, $50,000–$74,999, 75,000–99,999 and $100,000+.

Reported activities were classified by time spent engaged in work at their primary job (identified by ATUS tiered code 050101), sleep (identified by ATUS tiered code 010101, 010102 and 010199), and other non-work, non-sleep intensity-defined behaviors, specifically, sedentary behavior (identified by 1.0 to 1.5 METs) [10], [11], [12], light- (1.6 to 2.9 METs) [5], moderate- (3.0 to 5.9 METs) [10], [13], and vigorous-intensity (≥6.0 METs activities) [10], [13]. Undefined time was otherwise aggregated as unaccounted time. Together, all time-based variables added up to account for the full 24-hour (1,440 minutes) period. However, since only subsamples of the population are known to engage in any amount of moderate and vigorous intensity activities [1], [5], and resulting estimates are thus distorted by an overwhelming number of respondents who do none of these types of activity [5], we also consolidated all intensities (light through vigorous) of non-work physical activities.

CPS-reported occupations and linked MET values [5] were used to categorize respondents and further classify time spent working at primary jobs as sedentary occupations or non-sedentary occupations. A “sedentary occupation” (e.g., one typically characterized by the universe of office-type administrative work) is not precisely the same as a “sedentary behavior” (stoically sitting, e.g., seated reading), and likely includes at least some light activity (e.g., walking to department meetings, filing, standing to greet office visitors, etc.). Since the occupational MET crosswalk [10] considered stereotypical workday body position (sit, stand, walk, heavy labor) and intensity (light, moderate, vigorous) in the process of allocating summary MET values, the rare floor value possible was 1.5 METs. Less restrictively, we have previously identified “sedentary occupations” as those assigned values of <2.0 METs [5], and we employ that same convention here. To be clear, “sedentary occupations” are characterized by much time spent in “sedentary behaviors” but also plausibly at least some time in light intensity activities and therefore the exact MET values to identify “sedentary behaviors” (1.0 to 1.5 METs) and “sedentary occupations” (<2.0 METs) are not exactly the same.

### Analysis

Constructed sampling weights were used by following the online documentation (http://www.bls.gov/tus/ehdatafiles.htm) to ensure representativeness of the U.S. non-institutionalized civilian population. Descriptive analysis included means (± standard error, SE) and frequencies (weighted %), as appropriate, for descriptive characteristics (sex, age, race/ethnicity, BMI and household income), employment in sedentary occupation (yes or no), and reported time spent in work, sleep, sedentary behaviors, and light, moderate, and vigorous intensity activities (these last three separately and combined as a “total non-work physical activity” category). We tested for differences in all time-based variables by sex and dichotomy of employment in sedentary/non-sedentary occupations. Finally, we employed logistic and linear regression to examine the association between BMI and employment in a sedentary occupation, accounting for time spent in sleep, other non-work time spent in sedentary behaviors, and light, moderate, and vigorous intensity activities, sex, age, race/ethnicity and household income. All statistical analyses were conducted using SAS 9.3 (SAS institute, N.C.).

## Results

Since none of the findings reported below differed between occupation intensity categories classified using either summary or detailed approaches to linking MET values [10], we present the results using only the summary values. Descriptive characteristics of the analysis sample (total sample and stratified by sex) are presented in Table 1 and Table 2. Despite apparent mean differences (e.g., men working longer than women, women engaged in more light intensity physical activity than men), there were no statistically significant differences between men and women (i.e., no p<0.05) in any of the time-based variables.

**Table 1:** Descriptive Characteristics (frequencies and percents) for 20 to 64 Year-Olds Who Worked at Least 7 Hours during the Sampled Day: ATUS Eating & Health Module Data, 2006 to 2008 (n = 4,092).

Characteristic		Total	Men	Women
		n (%)^a^	n (%)^a^	n (%)^a^
		4092 (100)	2153 (55.7)	1939 (44.3)
Age, years				
	<30	643 (23.3)	326 (22.2)	317 (24.6)
	30–39	1178 (24.8)	648 (27.0)	530 (22.1)
	40–49	1250 (26.7)	673 (26.4)	577 (27.2)
	50–64	1021 (25.2)	506 (24.4)	515 (26.2)
Race/ethnicity				
	White- Hispanic	397 (9.8)	226 (11.5)	171 (7.7)
	White- non-Hispanic	3034 (75.2)	1638 (75.4)	1396 (74.9)
	Black-Hispanic	10 (0.3)	5 (0.4)	5 (0.2)
	Black- non-Hispanic	455 (9.5)	188 (8.2)	267 (11.2)
	Asian	114 (3.1)	58 (2.6)	56 (3.6)
	Other	82 (2.1)	38 (1.9)	44 (2.4)
BMI, kg/m^2^				
	18.50–24.99	1403 (34.8)	536 (25.0)	867 (47.0)
	25.00–27.49	894 (22.7)	571 (27.3)	323 (16.8)
	27.50–29.99	680 (16.1)	427 (18.6)	253 (12.9)
	30.00–34.99	724 (17.4)	430 (20.6)	294 (13.5)
	≥35.00	391 (9.1)	189 (8.4)	202 (9.8)
Household income				
	<$25,000	458 (9.7)	209 (9.9)	249 (9.4)
	$25,000–$49,999	1077 (24.0)	544 (24.7)	533 (23.2)
	$50,000–$74,999	918 (22.7)	472 (21.4)	446 (24.4)
	$75,000–$99,999	672 (17.5)	354 (16.1)	318 (19.2)
	$100,000+	967 (26.1)	574 (27.9)	393 (23.8)
Occupation				
	Sedentary	1718 (42.2)	845 (39.3)	873 (45.8)
	Non-sedentary	2374 (57.8)	1308 (60.7)	1066 (54.2)
				

a % = Weighted %.

**Table 2:** Descriptive Characteristics (Means and Standard Errors) for 20 to 64 Year-Olds Who Worked at Least 7 Hours during the Sampled Day: ATUS Eating & Health Module Data, 2006 to 2008 (n = 4,092).

Time, minutes	Mean ± SE^b^	Mean ± SE^b^	Mean ± SE^b^
Work	547.7±13.8	563.5±19.9	527.8±15.1
Non-work:			
Sleep	441.7±11.6	436.9±15.9	447.7±16.4
Sedentary behavior	155.2±13.9	159.0±18.8	150.4±19.5
Light intensity	263.8±15.3	245.5±20.3	287.0±20.5
Moderate intensity	24.2±5.6	27.3±8.2	20.4±6.4
Vigorous intensity	3.3±2.1	4.2±3.4	2.3±1.8
Undefined	4.0±2.0	3.7±2.9	4.4±3.0

b SE = Standard error.

**Figure 2:**
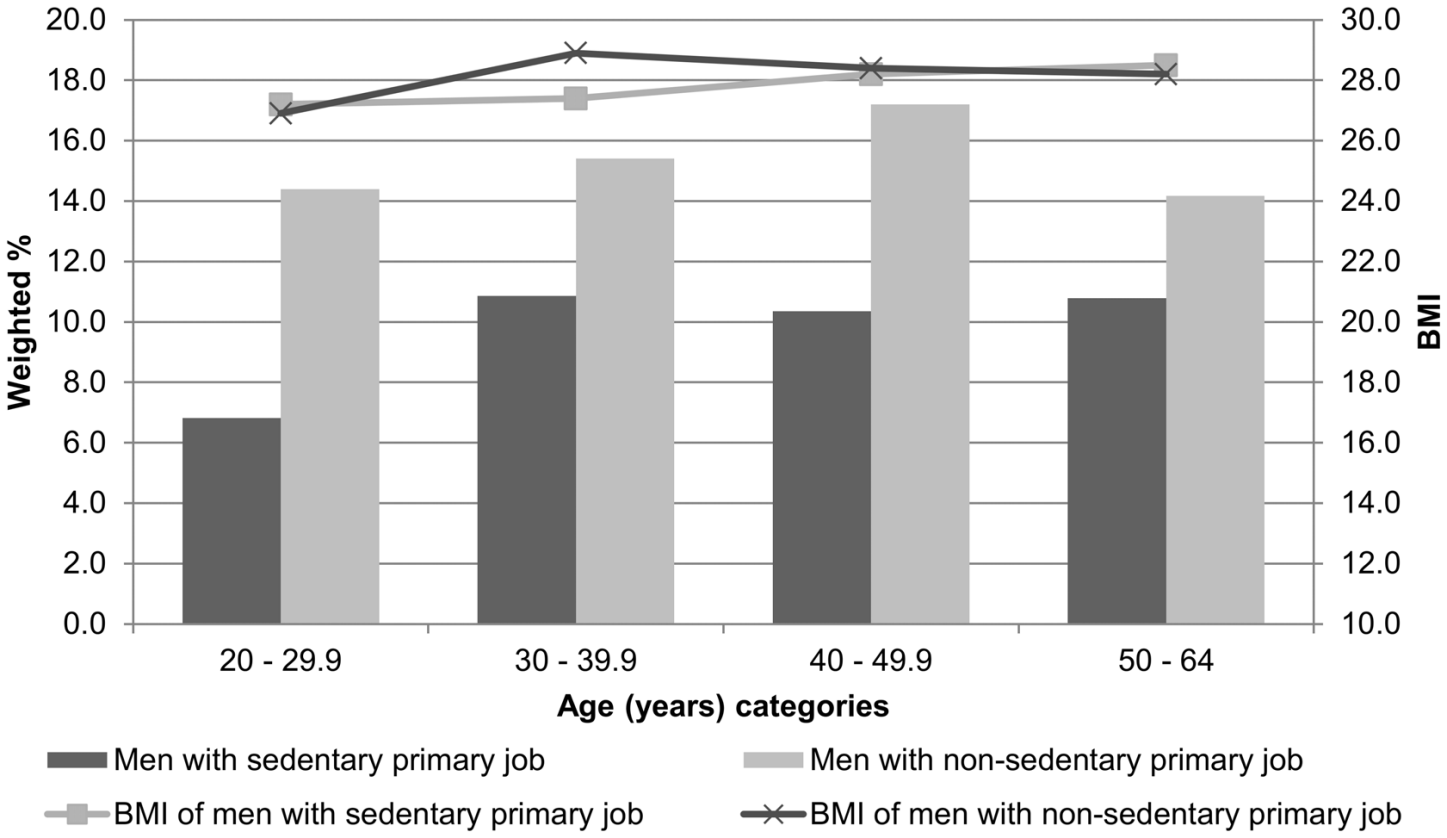
Weighted population percent and mean BMI of male respondents ages 20–64 years who worked ≥7 hours at their primary job during the sampled day: American Time Use Survey, Current Population Survey, Eating and Health Module 2006 to 2008 (n = 2,153).

**Figure 3:**
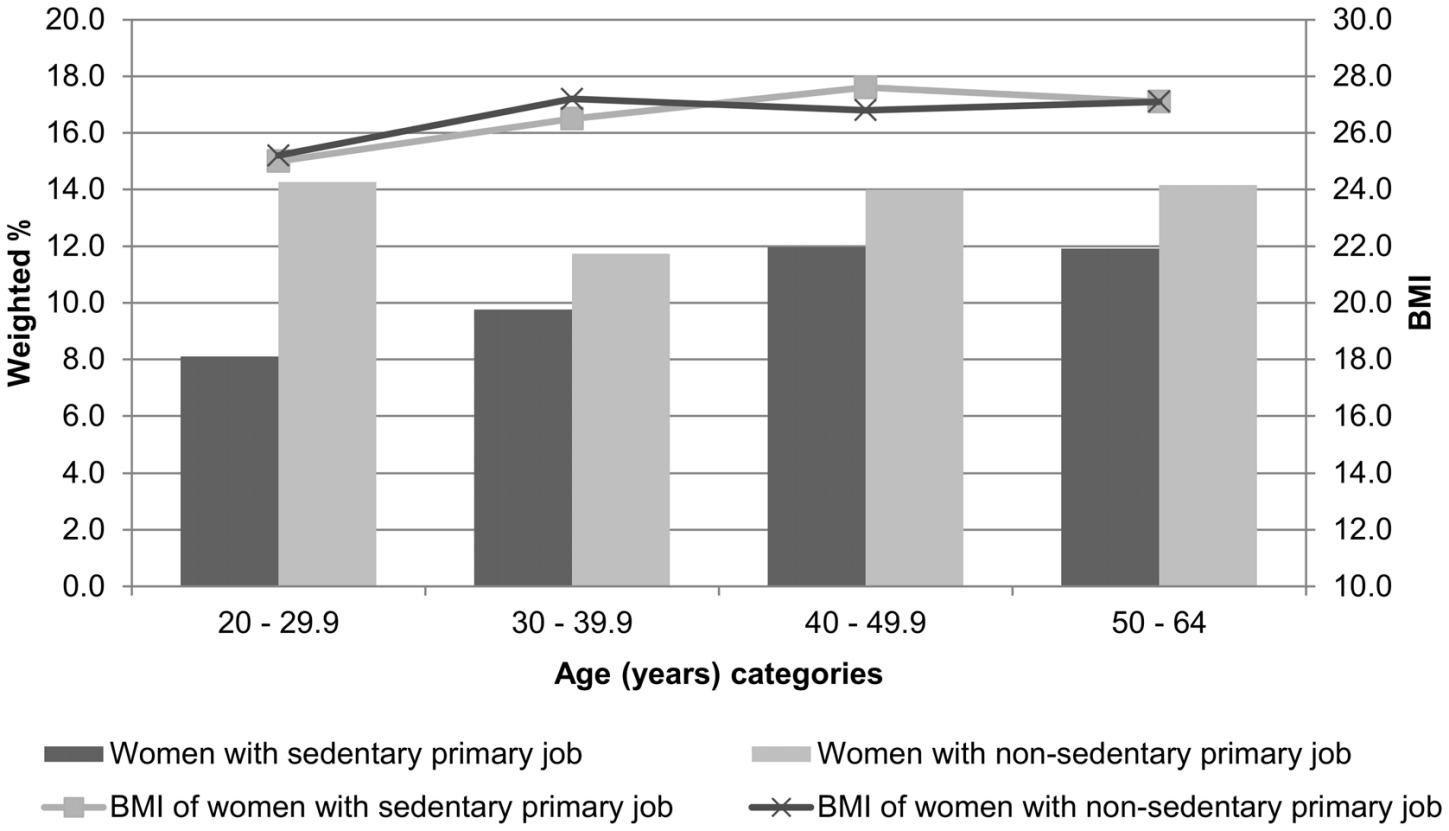
Weighted population percent and mean BMI of female respondents ages 2064 years and worked ≥7 hours at their primary job during the sampled day: American Time Use Survey, Current Population Survey, Eating and Health Module 2006 to 2008 (n = 1,939).

**Figure 4:**
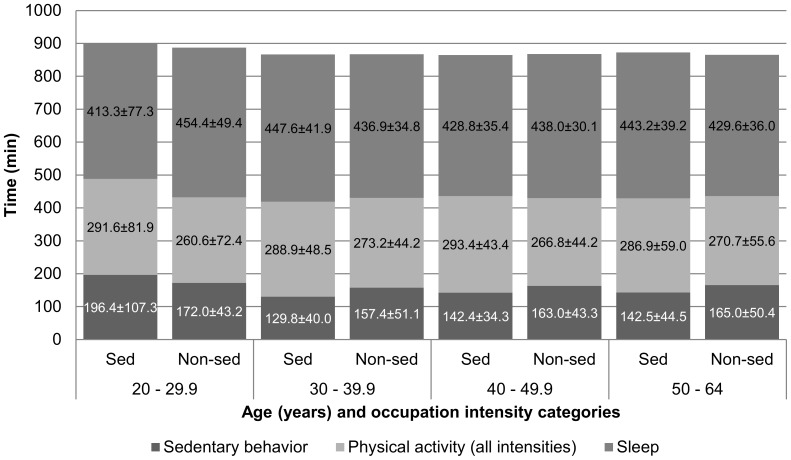
Male respondents' (ages 20–64 years, reported working ≥7 hours at their primary job during the sampled day) mean time (minutes ± SEM) spent in non-work sleep, sedentary behavior or physical activity (all intensities) by occupation intensity category (sedentary vs. non-sedentary): American Time Use Survey, Current Population Survey, Eating and Health Module 2006 to 2008 (n = 2,153).

**Figure 5:**
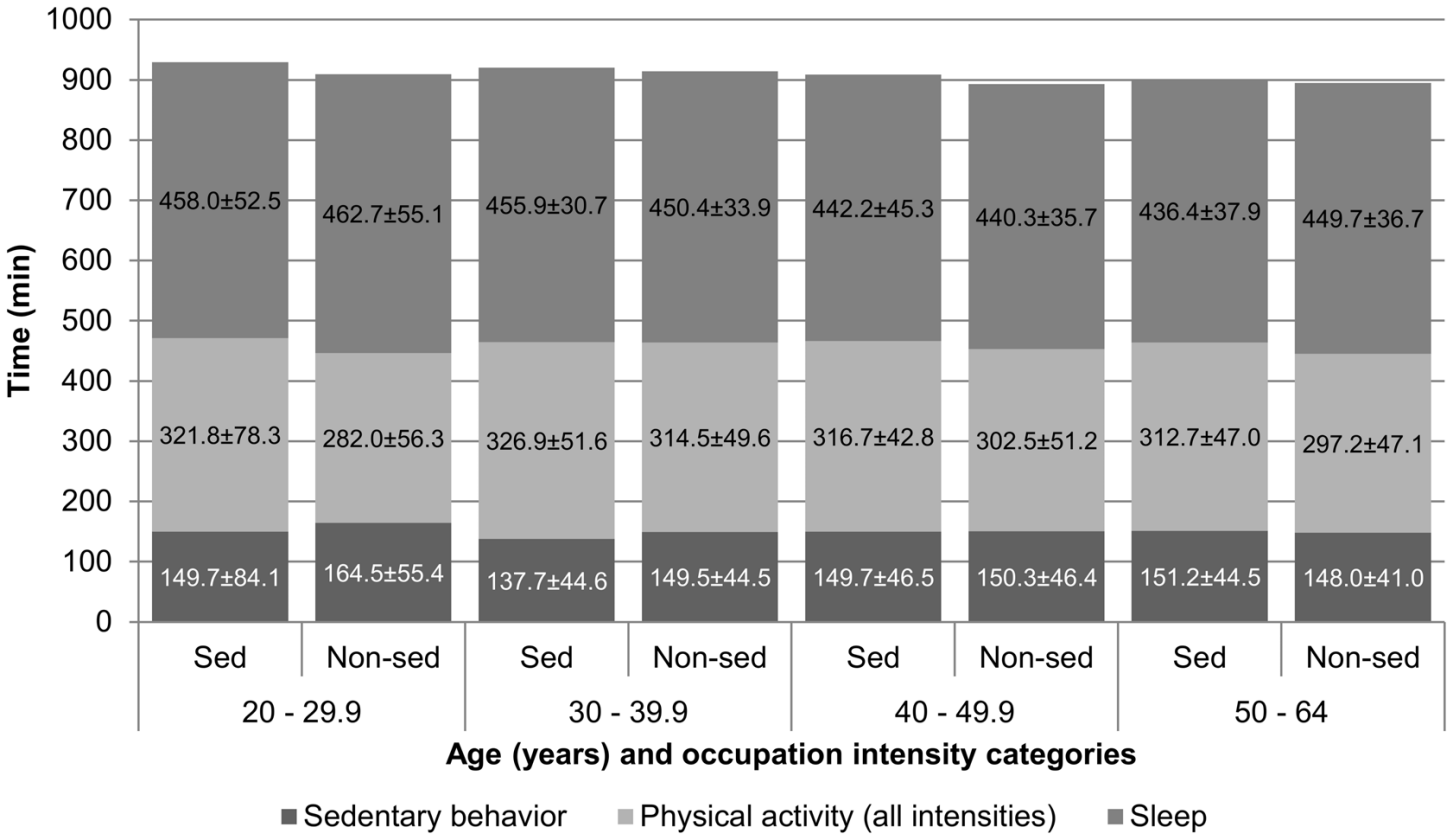
Female respondents' (ages 20–64 years, reported working ≥7 hours at their primary job during the sampled day) mean time (minutes ± SEM) spent in non-work sleep, sedentary behavior or physical activity (all intensities) by occupation intensity category (sedentary vs. non-sedentary): American Time Use Survey, Current Population Survey, Eating and Health Module 2006 to 2008 (n = 1,939).

The average male respondent employed in a sedentary occupation was 41.1±3.2 years of age and had a BMI of 27.9±1.2 kg/m^2^. His peer employed in a non-sedentary occupation was 39.6±2.7 years of age, and had a BMI of 28.1±1.2 kg/m^2^. Corresponding values for female respondents were 41.2±3.3 years of age (vs. 40.0±2.8 years of age), and a BMI of 26.7±1.7 kg/m^2^ (vs. 26.7±1.6 kg/m^2^). There were no statistically significant differences in age or BMI by sedentary/non-sedentary occupation dichotomy for either men or women. Likewise, there were no statistically significant differences in any of the time-based variables by sedentary/non-sedentary occupation dichotomy for men or women (Table 3). The weighted percent of the population and their mean BMI are presented by sedentary/non-sedentary occupation dichotomy and age groups for men (Figure 2) and women (Figure 3). Time spent in sleep, in other non-work sedentary behaviors, and in all non-work physical activities (light-, moderate-, and vigorous-intensity combined) are also presented by sedentary/non-sedentary occupation dichotomy for men (Figure 4) and women (Figure 5).

**Table 3:** Time (minutes, mean ± standard error) at work and non-work time by dichotomy of occupation intensity (sedentary/non-sedentary) within sex (n = 4,092).

Time (min)	Men		Women	
	Sedentary	Non-sedentary	Sedentary	Non-sedentary
Work	561.4±27.0	564.8±26.6	521.9±18.6	532.8±21.8
Sleep	435.5±24.3	437.8±19.3	446.9±21.0	448.4±24.7
Sedentary	148.4±28.7	165.9±25.5	146.6±29.4	153.7±25.1
Light	258.9±28.2	236.8±28.8	300.3±27.9	275.7±26.9
Moderate	26.6±12.2	27.7±10.8	17.2±7.3	23.2±9.9
Vigorous	5.3±6.3	3.5±3.8	2.5±2.8	2.1±2.6
Undefined	4.0±3.5	3.5±4.5	4.6±4.8	4.2±4.1

N.B. No significant differences in any time-based variables by dichotomy of occupation intensity (sedentary/non-sedentary) for men or women.

Logistic and linear regression analyses failed to reveal any associations between BMI and the sedentary/non-sedentary occupation dichotomy considering time spent in sleep, other non-work time spent in sedentary behaviors, and light, moderate, vigorous and undefined intensity activities, sex, age, race/ethnicity and household income (Tables 4 and 5).

**Table 4:** Linear regression analysis investigating the association between BMI and occupation intensity (sedentary/non-sedentary) of primary job (n = 4,092).

	Total			Male			Female		
Model^a^	B	SE	R^2^	B	SE	R^2^	B	SE	R^2^
0	−0.15	1.34	<.01	−0.18	1.68	<.01	0.07	2.43	<.01
1	0.17	1.38	0.02	−0.10	1.75	<.01	0.35	2.47	0.03
2	0.20	1.40	0.03	−0.07	1.76	0.01	0.33	2.57	0.04

B  = estimate of β-coefficient.

SE = standard error.

a Model 0 is simple linear regression;

Model 1 was adjusted for sex, age, race/ethnicity and household income;

Model 2 was adjusted for sex, age, race/ethnicity, household income and time spent in sleep, other non-work time spent in sedentary behaviors, and light, moderate, vigorous, and undefined intensity activities;

P-value of 3 models ranged from 0.89 to 0.98.

**Table 5:** Logistic regression analysis investigated the association between BMI and occupation intensity (sedentary/non-sedentary) of primary job (n = 4,092).

	Model 0^a^		Model 1^a^		Model 2^a^	
BMI	OR (95% CI)	P	OR (95% CI)	P	OR (95% CI)	P
18.5–24.9	1.00		1.00		1.00	
25.0–27.4	1.02 (0.26–4.03)	0.97	0.96 (0.23–3.93)	0.95	0.94 (0.23–3.91)	0.94
27.5–29.9	1.05 (0.25–4.45)	0.94	0.98 (0.21–4.69)	0.98	0.97 (0.20–4.65)	0.97
30.0–34.9	1.11 (0.30–4.07)	0.88	0.96 (0.25–3.67)	0.96	0.95 (0.24–3.69)	0.94
≥35.0	1.14 (0.20–6.51)	0.88	0.98 (0.16–5.92)	0.98	0.96 (0.15–5.97)	0.96
Male						
18.5–24.9	1.00		1.00		1.00	
25.0–27.4	0.97 (0.15–6.23)	0.98	1.05 (0.14–7.77)	0.96	1.03 (0.14–7.60)	0.98
27.5–29.9	1.01 (0.12–8.37)	1.00	1.07 (0.12–9.87)	0.96	1.06 (0.12–9.77)	0.96
30.0–34.9	1.05 (0.17–6.42)	0.96	1.06 (0.16–7.07)	0.95	1.04 (0.15–7.12)	0.97
≥35.0	1.21 (0.08–17.74)	0.89	1.18 (0.07–19.34)	0.91	1.16 (0.07–19.29)	0.92
Famale						
18.5–24.9	1.00		1.00		1.00	
25.0–27.4	0.93 (0.11–7.61)	0.95	0.91 (0.11–7.36)	0.93	0.92 (0.11–7.67)	0.94
27.5–29.9	0.98 (0.10–9.81)	0.98	0.95 (0.09–9.91)	0.97	0.94 (0.09–10.06)	0.96
30.0–34.9	1.03 (0.12––8.63)	0.98	0.93 (0.11–8.09)	0.94	0.94 (0.10–8.62)	0.95
≥35.0	1.03 (0.07–14.52)	0.99	0.88 (0.06–13.73)	0.93	0.88 (0.05–15.56)	0.93

OR (95%CI)  = odds ratio (95% confident interval weighted).

P = p-value.

a Model 0 is simple logistic regression;

Model 1was adjusted for sex, age, race/ethnicity and household income;

Model 2 was adjusted for sex, age, race/ethnicity, household income and time spent in sleep, other non-work time spent in sedentary behaviors, and light, moderate, vigorous and undefined intensity activities.

## Discussion

Previous surveys of Polish [14] (n = 508) and Spanish [15] (n = 12,044) workers did not find any significant associations between occupational energy expenditure and BMI after accounting for time spent in leisure time physical activities. Limiting survey design and subsequent analysis only to self-reported sedentary work and leisure-time physical activity implies that these two behaviors are the only variables worth tracking in relation to BMI. It stands to reason, however, that a complete inventory of obligatory and discretionary behaviors accumulated throughout the day interact to contribute to energy balance. In this analysis of the merged ATUS, CPS, and EH data sets, however, we found no evidence of a relationship between self-reported full time sedentary occupation classification and BMI after accounting for sex, age, race/ethnicity, household income, and 24-hours of time use including non-work related physical activity and sedentary behaviors.

Zick et al. [16] previously used the 2006–2007 ATUS and associated EH modules to conduct a multivariable analysis of the time use relationship with BMI. Although it is possible to account for all behaviors in the full day using these ATUS data as we have done, these authors applied our own MET classification [3] but focused only on time (specifically bouts of ≥10 minutes) accumulated over 3.3 METs as an indicator of activity, and only television/video viewing and time spent sleeping as indicators of inactivity. Further, although ATUS captures respondents' reported time spent at work, these authors' analytical treatment of this behavior was limited to the application of a dummy variable only in the case of males employed in occupations that were considered representative of work intensities greater than 3.3 METs. The authors did not seem to have considered the amount of time spent at work (i.e., full time workers) or the reduced energy expenditure associated with sedentary occupations, although these are much more common than moderate-to-vigorous intensity occupations [10]. Overall, their data reduction and analytical approach implies preconceived valuation of factors related to BMI, as opposed to a more inductive approach to identifying emergent factors from amongst the data. Our own approach was more exploratory, querying how BMI varies across full time occupations categorized as sedentary/non-sedentary, with and without accounting for a full day's contingent of time spent in sleep, other sedentary behaviors, and light, moderate, and vigorous intensity activities. The fact that we did not see a clear relationship between BMI and sedentary/non-sedentary occupations suggests that occupation alone is not a defining factor in shaping this health indicator. Our study supports a review that concluded that at this time there is limited evidence to substantiate a positive relationship between occupational sitting and health risks [17].

There are alternative explanations, however, for why we did not find such evidence. The ATUS, CPS, and EH are self-reported surveys, and although time use surveys have been validated against accelerometers [18], the assignment of MET codes representing intensity of reported behaviors and simplified occupational classification is admittedly inexact [3], [10]. Further, there is a tendency for adults to under-report weight and over-report height (both necessary to compute BMI) [19], although the vagaries in self-reported height and weight are likely more complicated than this generalized statement of reporting bias implies [20], [21]. That said, as an illustrative example of the potential for self-report bias to affect BMI measures, the prevalence of obesity (derived from self-reported height and weight) among women depicted herein (22.6%) is dramatically lower than nationally representative estimates of objectively measured obesity among adults (≥20 years) during the 2005–2006 and 2007–2008 National Health and Nutrition Examination Survey (35.3% and 35.5%, respectively) [22]. Such a large discrepancy between U.S. nationally representative estimates of self-reported and objectively measured obesity highlights the incongruity between these measures and the potential for self-report bias to introduce substantial amounts of measurement error. The EH module also queries time spent eating as a primary and secondary behavior, but this is not the same as querying what was consumed in terms of energy intake, so it is possible also that energy intake systematically differs in some way between occupational categories and confounds relationships with BMI. All told, it is plausible that these various sources of error could compound to obscure any real relationships.

In summary, we found no evident relationship between self-reported full time sedentary occupation classification and BMI after accounting for time spent in non-work related physical activity and sedentary behaviors, sex, age, race/ethnicity, and household income. Future analyses should focus on 24-hour objectively monitored behavior and directly assessed BMI.

## References

[1] Troiano RP, Berrigan D, Dodd KW, Masse LC, Tilert T, et al (2008) Physical activity in the United States measured by accelerometer. Medicine and Science in Sports and Exercise 40: 181–188. 18091006 10.1249/mss.0b013e31815a51b3

[2] Tudor-Locke C, Johnson WD, Katzmarzyk PT (2011) U.S. population profile of time-stamped accelerometer outputs: impact of wear time. Journal of Physical Activity and Health 8: 693–698. 21734315 10.1123/jpah.8.5.693

[3] Tudor-Locke C, Washington TL, Ainsworth BE, Troiano RP (2009) Linking the American Time Use Survey (ATUS) and the Compendium of Physical Activities: Methods and rationale. Journal of Physical Activity and Health 6: 347–353. 19564664 10.1123/jpah.6.3.347

[4] Van Domelen DR, Koster A, Caserotti P, Brychta RJ, Chen KY, et al (2011) Employment and physical activity in the U.S. Am J Prev Med. 41: 136–145. 21767720 10.1016/j.amepre.2011.03.019 PMC5221416

[5] Tudor-Locke C, Leonardi C, Johnson WD, Katzmarzyk PT (2011) Time spent in physical activity and sedentary behaviors on the working day: the American time use survey. J Occup Environ Med 53: 1382–1387. 22104979 10.1097/JOM.0b013e31823c1402

[6] Church TS, Thomas DM, Tudor-Locke C, Katzmarzyk PT, Earnest CP, et al (2011) Trends over 5 decades in U.S. occupation-related physical activity and their associations with obesity. PLoS ONE 6: e19657. 21647427 10.1371/journal.pone.0019657 PMC3102055

[7] Mummery WK, Schofield GM, Steele R, Eakin EG, Brown WJ (2005) Occupational sitting time and overweight and obesity in Australian workers. Am J Prev Med 29: 91–97. 16005804 10.1016/j.amepre.2005.04.003

[8] Monda KL, Adair LS, Zhai F, Popkin BM (2008) Longitudinal relationships between occupational and domestic physical activity patterns and body weight in China. Eur J Clin Nutr 62: 1318–1325. 17637599 10.1038/sj.ejcn.1602849

[9] Allman-Farinelli MA, Chey T, Merom D, Bauman AE (2010) Occupational risk of overweight and obesity: an analysis of the Australian Health Survey. J Occup Med Toxicol 5: 14. 20550716 10.1186/1745-6673-5-14 PMC2894850

[10] Tudor-Locke C, Ainsworth BE, Washington TL, Troiano RP (2011) Assigning metabolic equivalent (MET) values to the 2002 Census Occupational Classification System. Journal of Physical Activity and Health 8. 10.1123/jpah.8.4.581 21597131

[11] Owen N, Leslie E, Salmon J, Fotheringham MJ (2000) Environmental determinants of physical activity and sedentary behavior. Exerc Sport Sci Rev 28: 153–158. 11064848

[12] Pate RR, O'Neill JR, Lobelo F (2008) The evolving definition of “sedentary”. Exerc Sport Sci Rev 36: 173–178. 18815485 10.1097/JES.0b013e3181877d1a

[13] Physical Activity Guidelines Advisory Committee (2008) Physical Activity Guidelines Report, 2008. Washington, DC: U.S. Department of Health and Human Services,.

[14] Kaleta D, Makowiec-Dabrowska T, Jegier A (2007) Occupational and leisure-time energy expenditure and body mass index. Int J Occup Med Environ Health 20: 9–16. 17509966 10.2478/v10001-007-0009-1

[15] Gutierrez-Fisac JL, Guallar-Castillon P, Diez-Ganan L, Lopez Garcia E, Banegas BanegasJR, et al (2002) Work-related physical activity is not associated with body mass index and obesity. Obes Res 10: 270–276. 11943836 10.1038/oby.2002.37

[16] Zick CD, Stevens RB, Bryant WK (2011) Time use choices and healthy body weight: a multivariate analysis of data from the American Time Use Survey. Int J Behav Nutr Phys Act 8: 84. 21810246 10.1186/1479-5868-8-84 PMC3199736

[17] van Uffelen JG, Wong J, Chau JY, van der Ploeg HP, Riphagen I, et al (2010) Occupational sitting and health risks: a systematic review. Am J Prev Med 39: 379–388. 20837291 10.1016/j.amepre.2010.05.024

[18] van der Ploeg HP, Merom D, Chau JY, Bittman M, Trost SG, et al (2010) Advances in population surveillance for physical activity and sedentary behavior: reliability and validity of time use surveys. Am J Epidemiol 172: 1199–1206. 20855469 10.1093/aje/kwq265

[19] McAdams MA, Van Dam RM, Hu FB (2007) Comparison of self-reported and measured BMI as correlates of disease markers in US adults. Obesity (Silver Spring) 15: 188–196. 17228047 10.1038/oby.2007.504

[20] Villanueva EV (2001) The validity of self-reported weight in US adults: a population based cross-sectional study. BMC Public Health 1: 11. 11716792 10.1186/1471-2458-1-11 PMC59896

[21] Gillum RF, Sempos CT (2005) Ethnic variation in validity of classification of overweight and obesity using self-reported weight and height in American women and men: the Third National Health and Nutrition Examination Survey. Nutr J 4: 27. 16209706 10.1186/1475-2891-4-27 PMC1262765

[22] Ogden CL, Carroll MD (2010) Prevalence of overweight, obesity, and extreme obesity among adults: United States, trends 1960–1962 through 2007–2008. National Vital Statistics Report 6: 1–10.

