# Correction: Body Mass Index: Accounting for Full Time Sedentary Occupation and 24-Hr Self-Reported Time Use

**DOI:** 10.1371/journal.pone.0118117

**Published:** 2015-03-06

**Authors:** The PLOS ONE Staff

The following information is missing from the Competing Interests section: "The views expressed are those of Dr. Hamrick’s, and should not be attributed to the Economic Research Service or USDA. "The correct competing interest information is as follows.

Dr. Hamrick, one of the co-authors, is employed by the USDA Economic Research Service. The views expressed are those of Dr. Hamrick’s, and should not be attributed to the Economic Research Service or USDA. There are no patents, products in development or marketed products to declare. This does not alter the authors' adherence to all PLOS ONE policies on sharing data and materials.

## References

[1] Tudor-Locke C, Schuna JM Jr, Katzmarzyk PT, Liu W, Hamrick KS, et al (2014) Body Mass Index: Accounting for Full Time Sedentary Occupation and 24-Hr Self-Reported Time Use. PLoS ONE 9(10): e109051 doi:10.1371/journal.pone.0109051 25295601 10.1371/journal.pone.0109051 PMC4189931

